# Ultrafast transient sub-bandgap absorption of monolayer MoS_2_

**DOI:** 10.1038/s41377-021-00462-4

**Published:** 2021-01-29

**Authors:** Susobhan Das, Yadong Wang, Yunyun Dai, Shisheng Li, Zhipei Sun

**Affiliations:** 1 Department of Electronics and Nanoengineering, Aalto University, 02150 Espoo, Finland; 2 International Center for Young Scientists (ICYS), National Institute for Materials Science (NIMS), Tsukuba, Japan; 3 QTF Centre of Excellence, Department of Applied Physics, Aalto University, Espoo, Finland

**Keywords:** Physical sciences, Physics, Optical physics, Ultrafast photonics

## Abstract

The light–matter interaction in materials is of remarkable interest for various photonic and optoelectronic applications, which is intrinsically determined by the bandgap of the materials involved. To extend the applications beyond the bandgap limit, it is of great significance to study the light–matter interaction below the material bandgap. Here, we report the ultrafast transient absorption of monolayer molybdenum disulfide in its sub-bandgap region from ~0.86 µm to 1.4 µm. Even though this spectral range is below the bandgap, we observe a significant absorbance enhancement up to ~4.2% in the monolayer molybdenum disulfide (comparable to its absorption within the bandgap region) due to pump-induced absorption by the excited carrier states. The different rise times of the transient absorption at different wavelengths indicate the various contributions of the different carrier states (i.e., real carrier states in the short-wavelength region of ~<1 µm, and exciton states in the long wavelength region of ~>1 µm). Our results elucidate the fundamental understanding regarding the optical properties, excited carrier states, and carrier dynamics in the technologically important near-infrared region, which potentially leads to various photonic and optoelectronic applications (e.g., excited-state-based photodetectors and modulators) of two-dimensional materials and their heterostructures beyond their intrinsic bandgap limitations.

## Photonics: Lighting the way to new photonic and optoelectronic applications

Light–matter interactions in two-dimensional (2D) materials are of interest for applications such as lasers and solar cells, but these interactions drastically decrease below the bandgap. Recently, phenomena in the sub-bandgap region of 2D materials have attracted attention, but the physics behind is still elusive. A team of researchers, led by Zhipei Sun from Aalto University in Finland, has studied the near-infrared (NIR) transient absorption of monolayer molybdenum disulfide in the sub-bandgap region. The work has provided a better understanding of optical properties, excited carrier states, and carrier dynamics in the technologically important NIR region, paving the way for excited-state-based photodetectors and modulators made from 2D materials.

## Introduction

The light–matter interaction in materials is fundamental to various photonic and optoelectronic applications, such as lasers, modulators and solar cells, which are intrinsically determined by the bandgap of the materials. When the photon energy is larger than the bandgap of a material, the light–matter interaction is typically stronger. This is particularly true for various recently discovered nanomaterials. For example, two-dimensional (2D) layer materials of transition metal dichalcogenides (TMDs) have been demonstrated as potential candidates for extremely strong light–matter interactions within their bandgap region despite their few-atomic thicknesses. Since monolayer and few-layer TMDs typically have bandgaps in the visible spectral range^1,2^, they are excellent candidates for ultrasensitive photodetectors^3^, ultrafast modulators^4,5^, lasers^6^, valleytronics^7^, and nonlinear optical devices^8–11^ in the visible spectral regime. On the other hand, it is well known that below the bandgap, the light–matter interaction decreases drastically. For example, the optical bandgap of monolayer molybdenum disulfide (ML-MoS_2_) is ~1.83 eV^12^; therefore, the absorbance of ML-MoS_2_ in the near-infrared (NIR) and longer spectral regions should be close to zero. However, very recently, it was demonstrated that the absorbance of ML-MoS_2_ in the sub-bandgap region can be very large (e.g., ~3% at 950 nm), possibly due to impurities, defects and edge states^12–15^. This phenomenon has attracted significant interest in pushing the applications of MoS_2_ and other TMDs in the technologically important NIR region and beyond, such as infrared detectors^16–18^ and saturable absorbers^6,19–28^ at the telecommunication wavelength range of ~1.5 µm and the mid-infrared wavelength region. To better understand these intriguing applications, it is of high importance to study the optical properties and the carrier relaxation dynamics of TMDs in the sub-bandgap region, which can fully extend the applications of TMDs beyond their bandgap limitation.

Here, we study the ultrafast transient absorption (TA) properties of ML-MoS_2_ flakes in the NIR region (i.e., from ~0.86 to 1.4 µm) and investigate their ultrafast carrier dynamics when excited by pump light at 400 nm in a reflection geometry. Although the probe wavelength range is smaller than the bandgap of ML-MoS_2_, ML-MoS_2_ absorption in this spectral range is enhanced in the presence of the pump light, reaching up to ~6% (with the maximum absorption enhancement of ~4.2%). Our TA measurements on ML-MoS_2_ provide insight into the carrier dynamics in the NIR region. Since the probe wavelength range covers the O-band and partially covers ~0.85 µm telecommunication windows, the experimental results reveal the behavior of the photo-excited carriers in ML-MoS_2_ below its bandgap and provide a fundamental understanding of its usability in the NIR spectral range for telecommunication applications.

## Results

**Fig. 1:**
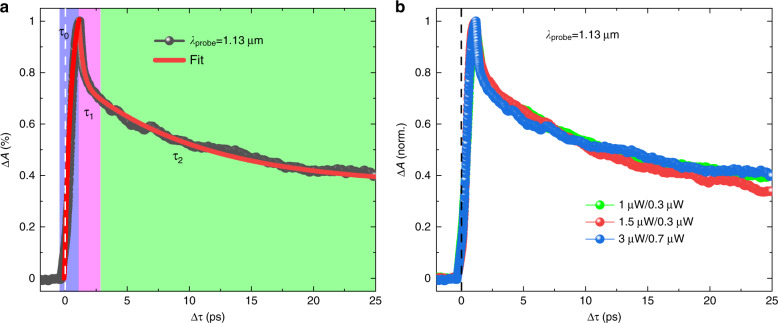
Transient absorption dynamics at a probe wavelength of ~1.13 µm. **a** Time-resolved absorption modulation when the average pump power *P*_*p*_ (intensity *I*_*p*_) is ~0.3 µW (~4.53 GW cm^-2^) and the average probe power *P*_*s*_ (intensity *I*_*s*_) is ~1.5 µW (~28.93 GW cm^−2^). An illustration of the rise time (*τ*_*0*_), fast recovery (*τ*_*1*_), and slow recovery (*τ*_*2*_) time constants is labeled. The rise and decay curves are fitted with single and bi-exponential functions, respectively. **b** Normalized time-resolved absorption modulation at different probe/pump powers. The zero-delay position is indicated in the Figures by the white and black dashed lines

A typical ultrafast TA at a wavelength far below the A-exciton of ML-MoS_2_ is shown in Fig. 1a (details of the experimental setup are given in the “Methods” section). Here, we use a probe wavelength of ~1.13 µm with an average power *P*_*s*_ (power intensity *I*_*s*_) of ~1.5 µW (~28.93 GW cm^−2^) while using a pump beam at a 400 nm wavelength with an average power *P*_*p*_ (power intensity *I*_*p*_) of ~0.3 µW (~4.53 GW cm^−2^). The absorption modulation at the probe wavelength from the reflection change is estimated by deploying the transfer matrix method^29,30^ based on our sample structure (details given in the Supplementary Information).

In Fig. 1a, the time-resolved absorption modulation (∆*A* = *A*_1_-*A*_0_) is calculated. *A*_1_ and *A*_0_ are the absorptions with and without the presence of the pump light. The delay time ∆*τ* is the relative time difference between the pump and probe pulses at the sample. The zero-delay position is precisely determined by the difference frequency generation process between the pump and probe pulses from the same ML-MoS_2_ flake and is indicated by the white dashed line in Fig. 1a. The results (Fig. 1a) clearly indicate TA modulation, which rises first and then decays.

In our reflection geometry of the pump-probe setup, since the probe energy is smaller than the bandgap of ML-MoS_2_, most of the probe light reflects back and remains unchanged without the presence of the pump beam. As the pump photon energy (~3.1 eV) is higher than the bandgap of ML-MoS_2_, the electrons from the valance band are excited to the quasi-continuum band by the pump beam. These excited electrons are commonly known to enhance the absorption of the probe light compared to unexcited ML-MoS_2_^31,32^. Note that we do not observe the dynamics on the bare substrate, which confirms that the TA is from the ML-MoS_2_ flake.

As shown in Fig. 1a, the increase in ∆*A* (highlighted in purple) from zero to its maximum value is very fast. In the presence of pump light, ∆*A* is immediately increased with a single-exponential rising time constant (*τ*_*0*_ = ~538 fs). Once the absorption modulation ∆*A* reaches its highest level, it starts to recover. At the beginning of the recovery, the intensity of ∆*A* drops down to a certain level very quickly (highlighted in magenta) and then decays slowly (highlighted in green). The decay dynamics can be fitted (red curve) by two exponential time constants (i.e., fast (*τ*_*1*_) and slow (*τ*_*2*_) recovery constants), and the different recovery domains are shown in Fig. 1a. At a probe wavelength of 1.13 µm in Fig. 1a, the fitted *τ*_*1*_ and *τ*_*2*_ are ~1.02 ps and ~80.5 ps, respectively. For further investigation of the carrier dynamics, we study the TA under various pump and probe powers (Fig. 1b). It is evident that the carrier dynamics are independent of both the pump and probe powers. Since the carrier dynamics do not exhibit a strong dependency on the pump and probe fluence, we can rule out the exciton-exciton annihilation process^33^.

**Fig. 2:**
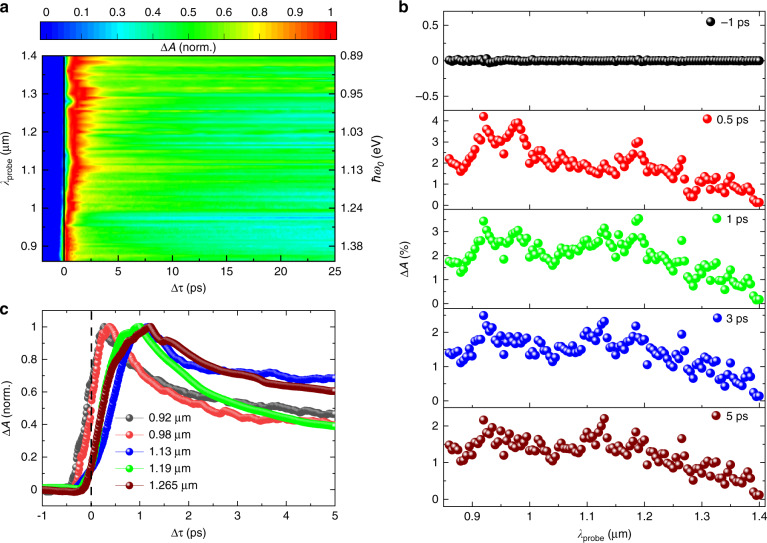
Broad transient absorption modulation in the NIR region. **a** Mapping of normalized absorption modulation (∆*A*) over the probe spectrum range from ~0.86 to 1.4 µm. The average pump power is ~0.3 µW, and the average probe power is ~1.5 µW. **b** TA modulation (∆*A*) at different delay times (∆*τ*). **c** Normalized TA modulation (∆*A*) at five selected probe wavelengths of ~0.92 (black), 0.98 (red), 1.13 (blue), 1.19 (green), and 1.265 µm (brown). The black dashed line indicates the zero-delay position

Furthermore, to explore the carrier dynamics in the whole NIR region, we tune the probe wavelength from ~0.86 to 1.4 µm on the same sample. The normalized time-resolved absorption modulation (∆*A*) mapping is shown in Fig. 2a, with an average probe power *P*_*s*_ ≃ 1.5 µW and average pump power *P*_*p*_ ≃ 0.3 µW. The zero-delay position is indicated by the black solid line in Fig. 2a. Owing to the presence of the pump light, the absorption of the probe beam in ML-MoS_2_ changes across the whole-probe spectrum range. From Fig. 2a, we can observe that within the proximity of the 1 µm probe wavelength, the increase in TA gradually extends to a longer delay time (i.e., the increase starts at ~−440 fs at ~0.88 µm and starts at ~−50 fs at ~1.3 µm). Details can also be found in Fig. S4.

Figure 2b displays the absorption modulation at different ∆*τ*. Before the arrival of the pump pulse, the absorption modulation ∆*A* over the whole-probe wavelength range is zero, as shown in the top panel of Fig. 2b. After the exposure of the pump beam, ∆*A* changes as ∆*τ* increases, as shown in the other panels of Fig. 2b. For the whole-probe spectrum, ∆*A* is positive when ∆*τ* > 0, which indicates the enhancement of the probe light absorption with the incidence of the pump beam. From Fig. 2b, it is also evident that under constant *P*_*s*_ and *P*_*p*_, ∆*A* varies at different probe wavelengths. As ∆*τ* increases, the peaks diminish and show overall broad-band modulation spectra, as shown in the last two panels of Fig. 2b. This observation signifies that even though the absorption modulation is a broad-band phenomenon, it is dependent on the probe wavelengths. Note that we observe modulation peaks at ~0.92 and 0.98 µm with more than ~4% absorption modulation and peaks at ~1.13, 1.19, and 1.265 µm with more than ~3% absorption modulation. This observation indicates that the absorption efficiency is comparatively higher at probe wavelengths in the presence of pump light. We assume that these peaks appear because of their energy matching of the carrier state transitions to the probe wavelength. The normalized time-resolved ∆*A* at the probe wavelengths corresponding to the peaks is shown in Fig. 2c. The results indicate that the ∆*τ* value for attaining maximum modulation varies. The peaks at ~0.92 and 0.98 µm attain the maximum level at ∆*τ* = *~*0.3 ps, whereas the peaks at ~1.13, 1.19, and 1.265 µm attain the maximum level at ∆*τ* = *~*1 ps. The details of the different time measurements, including time constants over the probe spectrum range, are given in the Supplementary Information. Therefore, here, we divide the whole-probe wavelength span (i.e., from ~0.86 to 1.4 µm) into two sections: shorter wavelength (~<1 µm) and longer wavelength (~>1 µm) ranges.

For the shorter probe wavelength range, the increase in ∆*A* is instantaneous and almost at the limit of our experimental time resolution. Therefore, exponential fitting is meaningless for that probe wavelength range. This ultrafast response is possibly a result of the ultrafast thermalization of extremely non-thermal carriers by carrier-carrier scattering^34–37^, which is assumed to be very quick due to the reduced screening effect in 2D materials. Figure 2c shows that for the longer probe wavelength range, the transient dynamics are quite different at the beginning. Furthermore, the increase in ∆*A* takes place in a longer delay time range (Fig. 2a, c). More specifically, at the modulation peak wavelengths of ~1.13, 1.19, and 1.265 µm, the rise time constant (*τ*_0_) is ~477 ± 80 fs. This result signifies that the photophysical mechanism for longer probe wavelengths is slower than that for shorter probe wavelengths. From the mathematical fitting, we find that the fast recovery time constant (*τ*_1_) is ~1.05 ± 0.39 ps for the whole-probe wavelength range. However, there is a slight increasing tendency of the fast recovery time constant (*τ*_1_) that is observed as the probe wavelength increases (Fig. S4a). However, the slow recovery time constant (*τ*_2_) does not have this tendency and sustains its value of ~93.9 ± 14 ps over the probe spectrum span. Possible mechanisms of the different decay processes are discussed later.

**Fig. 3:**
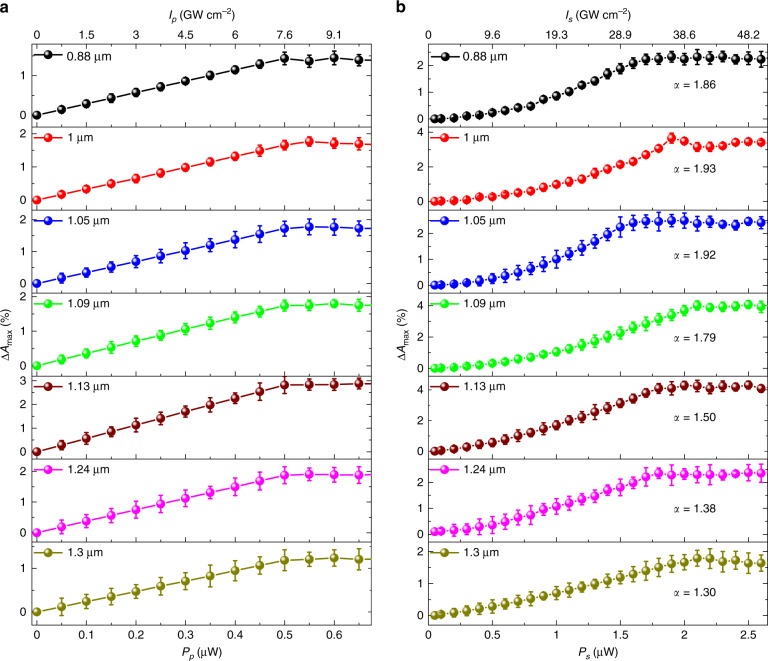
Power-dependent maximum absorption modulation (∆*A*_max_). ∆*A*_max_ at selected probe light wavelengths w.r.t. **a** the pump and **b** the probe light power with a fixed probe light power of ~1 µW and a fixed pump light power of ~0.3 µW, respectively

From the power-dependent study of TA dynamics at a few selective probe wavelengths, we reveal that the dynamics are independent of both the pump and probe power, the details of which are given in the Supplementary Information. The observed behavior is similar to that in Fig. 1b. Even though the TA dynamics are independent of *P*_*p*_ (*I*_*p*_) and *P*_*s*_ (*I*_*s*_), the maximum absorption modulation (∆*A*_max_) is highly influenced by them. We studied both *P*_*p*_ (*I*_*p*_)- and *P*_*s*_ (*I*_*s*_)-dependent ∆*A*_max_ by keeping *P*_*s*_ (*I*_*s*_) and *P*_*p*_ (*I*_*p*_) constant successively with an appropriate delay time ∆*τ*, where ∆*A* is at its maximum. The dependency of ∆*A*_max_ on *P*_*p*_ (*I*_*p*_) by keeping *P*_*s*_ (*I*_*s*_) fixed at ~1 µW (~19.29 GW cm^−2^) is shown in Fig. 3a for a few selected probe wavelengths. Before it reaches the saturation point, there is a linear relationship between ∆*A*_max_ and *P*_*p*_. As *P*_*p*_ increases, ∆*A*_max_ increases accordingly until saturation. It is evident that when *P*_*p*_ (*I*_*p*_) reaches ~0.5 µW (~7.55 GW cm^−2^) and beyond, ∆*A* becomes saturated and has no observable change within the error limit. Likewise, the influence of *P*_*s*_ (*I*_*s*_) on ∆*A*_max_ is studied with a fixed *P*_*p*_ (*I*_*p*_) of 0.3 µW (~4.53 GW cm^−2^) at different probe wavelengths, as depicted in Fig. 3b. The observed maximum value of ∆*A*_max_ can be as high as ~4.2% at the 1.13 µm probe wavelength. Here, we also observe the saturation effect at high *P*_*s*_ (*I*_*s*_). However, before the saturation of ∆*A*_max_, we observe a nonlinear relation between *P*_*s*_ and ∆*A*_max_. From the mathematical fitting, it can be concluded that

$${\Delta}A_{{\mathrm{max}}} \propto P_{p}\,{\mathrm{for}}\,P_{p} \, < \, P_{{\mathrm{p}}\_{\mathrm{sat}}}\,{\mathrm{at}}\,{\mathrm{fixed}}\,P_s$$

(Fig. 3a)

$${\Delta}A_{{\mathrm{max}}} \propto P_s^\alpha \,{\mathrm{for}}\,P_s \,<\, P_{{\mathrm{s}}\_{\mathrm{sat}}}\,{\mathrm{at}}\,{\mathrm{{{fixed}}}}\,P_p$$

(Fig. 3b), where *α* is the power law coefficient. *P*_p_sat_ and *P*_s_sat_ are the pump and probe power threshold levels, respectively, beyond which the absorption modulation saturates.

The dependency of ∆*A* on *P*_*p*_ (i.e., ${\Delta}A_{{\mathrm{max}}} \propto P_p$, Fig. 3a) means that the contribution of the pump beam is linear, indicating a one-photon excitation and contribution process (i.e., one absorbed pump photon creates one excited carrier and presents excited-state introduced absorption at the probe wavelength). As shown in Fig. 3a, saturation of the absorption modulation occurs while increasing the pump power under a fixed probe power (~1 µW). The pump is responsible for creating hot carriers in ML-MoS_2_. Therefore, the saturation in ∆*A*_max_ with increasing pump power in Fig. 3a is due to the Pauli blocking effect^24,38^. On the other hand, in Fig. 3b, the saturation of ∆*A*_max_ occurs with increasing probe power under a fixed pump power (~0.3 µW). As the number of hot electrons created by the pump beam in the system is fixed, the enhanced absorption at the probe wavelength can be limited by the existing hot carriers. Therefore, the saturation with increasing probe power in Fig. 3b can be attributed to the deficiency of hot carriers that are created by the pump light.

**Fig. 4:**
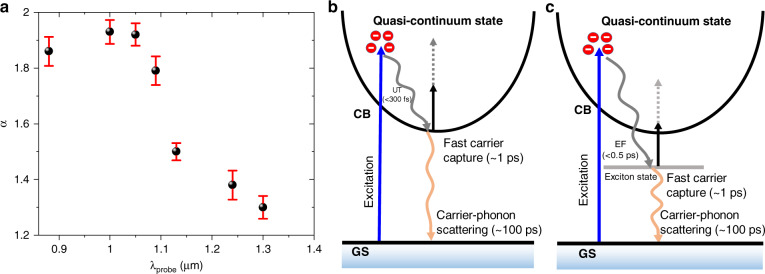
Photophysical phenomena in the NIR region. **a** Power law coefficient extracted from Fig. 3b at different probe wavelengths. An illustration of the TA dynamics with excitation in **b** the shorter probe wavelength range (~<1 µm) and **c** the longer probe wavelength range (~>1 µm). GS ground state, CB conduction band, UT ultrafast thermalization, EF exciton formation

The value of *α* at different probe wavelengths is shown in Fig. 3b. The dependency of ∆*A* on *P*_*s*_ (i.e., ${\Delta}A_{{\mathrm{max}}} \propto P_s^\alpha$) is not linear, which signifies that two-photon absorption (TPA) phenomena at the probe wavelength take place before it hits the saturation region. The value of *α* has a tendency to decrease with increasing probe wavelength, which indicates that the two-photon absorption is more dominant at shorter probe wavelengths and becomes more linear at longer wavelengths, as shown in Fig. 4a. This outcome is possibly due to the enhanced intra-band absorption at the longer wavelength of the excited carriers, which is expected to be linear.

## Discussion

Examining the dynamics at individual wavelengths of the NIR region associated with the features in the TA spectrum provides information about the time-scales of the different photophysical processes in ML-MoS_2_. Since the pulse width of both the pump and probe beams is ~230 fs, any photophysical phenomena with a time constant smaller than it can be inconclusive due to the constraint of the time resolution. Since the pump beam energy is far above the bandgap of ML-MoS_2_, the carriers from the ground state jump to the quasi-continuum state, as shown in Fig. 4b, c. The absorption at different probe wavelengths is influenced by these excited carriers generated in ML-MoS_2_. Since the absorption increases over the whole-probe wavelength range due to these excited states, this phenomenon can be simply treated as photoinduced absorption. The excited carriers imprint a relaxation pathway as the transient dynamics based on the probe wavelength. In our TA measurement, since the probe photon energy is much larger than the exciton binding energies and much smaller than the optical bandgap of ML-MoS_2_, excitons contribute to the intra-band absorption in approximately the same way as the free carriers^39–42^ but at different energy levels.

It has been reported that the electrical bandgap of ML-MoS_2_ is ~2.47 eV^43^. We thus correlate this with our results, as we observe strong two-photon absorption in the shorter wavelength range (<~1 µm, as shown in Fig. 4a). Therefore, we describe the possible phenomena with two different models for the shorter and longer wavelength regions, as shown in Fig. 4b, c, respectively. Furthermore, from the transient absorption dynamics shown in Fig. 2c, it is clear that the dynamics are different between the shorter wavelength range (<~1 µm) and the longer wavelength range (>~1 µm). From the rise time (t_rise_ from 1% to 99%) calculation (Fig. S4b), there is a clear indication of a break at an ~1 µm probe wavelength, which explains the boundary between the two models.

As we observed from our measurements, the rise time (*t*_rise_) at shorter probe wavelengths (<~1 µm) is instantaneous (*t*_rise_ = ~685 ± 75 fs in Fig. S4b, the time constant *τ*_0_ < 300 fs after fitting) and close to the experimental time resolution limit. Therefore, we attribute this phenomenon to the ultrafast thermalization of hot carriers via carrier-carrier scattering^34–37^, as shown in Fig. 4b. Tuning the probe beam from shorter to longer wavelengths also allows us to follow the subsequent carrier cooling of the carrier distribution into lower energy states. Therefore, a longer rise time is expected for a longer probe wavelength^44^. Additionally, we observe TPA at an ~0.92 µm probe wavelength with a TPA coefficient of ~5.83 × 10^3^ cm GW^−1^ (details given in the Fig. S6) and in the NIR region, similar to the previously reported large TPA results on ML-MoS_2_^45,46^. Therefore, considering the two-photon excitation with the probe photon energy, shorter wavelengths are close to the conduction band, whereas longer wavelengths are close to the exciton levels, which can be the possible reason for the distinction in the rising portion of the TA dynamics shown in Fig. 2c. Note that for the longer probe wavelength range, the long rise time (*t*_rise_ shown in Fig. S4b) is ~1.09 ± 08 ps (the rise time constant *τ*_0_ = ~477 ± 80 fs after fitting; see Fig. S4a), which possibly indicates exciton formation in ML-MoS_2_, as reported^47,48^. We therefore assume that the enhancement of absorption in this wavelength range of our experiment (>~1 µm) is due to the exciton formation, as shown in Fig. 4c.

For monolayer TMDs on a SiO_2_/Si substrate, essentially all the atoms are on the surface or at the interfaces. After ultrafast thermalization and the formation of excited states, as shown in Fig. 4b, c, respectively, the hot carriers relax to the ground state (GS), which indicates the decay dynamics. From the mathematical fitting, the recovery process can be described as a two-step process (details are given in the Supplementary Information). Since the carrier dynamics do not exhibit a strong dependency on the pump and probe fluence (Fig. 1b), we can rule out exciton-exciton annihilation processes. The initial fast recovery occurs within the time range of ~1.05 ± 0.39 ps, which possibly indicates both carrier-carrier scattering and fast carrier capture. However, for monolayer crystals, the carrier-carrier scattering response time is typically much faster (on the order of femtoseconds)^47,49^. Therefore, we can mainly attribute the fast recovery dynamics to fast carrier capture^36^. The invariant TA dynamics over the pump power (Fig. 1b) and linear increment of the absorption modulation ∆*A* (Fig. 4a) further ensure this process. The time constant for the slow recovery process (*τ*_2_) is on the order of tens of picoseconds (~93.9 ± 14 ps) irrespective of the probe wavelength; therefore, the slow recovery process can be mainly attributed to carrier-phonon scattering^37,50–52^. Therefore, our model fits well with the whole-probe wavelength range, and the time constant calculation strongly supports our proposed model. Similar TA dynamics on ML-WS_2_ (details given in the Supplementary Information) are observed, which also has good agreement with the proposed model.

In conclusion, we have studied the TA of ML-MoS_2_ in the NIR region from ~0.86 to 1.4 µm, discovering that the achieved enhancement of absorption can be up to 4.2%. We obtained temporally resolved carrier dynamics with a resolution of hundreds of femtoseconds, which provides a detailed view regarding the relaxation time of the carriers. Our findings ensure that the photophysical phenomena for the shorter wavelength range (<1 µm) are different from those for the longer wavelength range (>1 µm) in the NIR region. The measured rise time is higher in the longer wavelength range than in the shorter wavelength range, showing different photophysical processes. Furthermore, we observed that the absorption modulation amplitude is highly dependent on the pump and probe intensities. The results show that due to the presence of excited carrier states in ML-MoS_2_, one-photon and two-photon absorption processes take place simultaneously at different wavelengths. Interestingly, our results reveal that shorter wavelengths have higher efficiency for two-photon absorption than longer wavelengths in the NIR region, which potentially leads to various photonic and optoelectronic applications of 2D materials and their heterostructures beyond their intrinsic bandgap limitation (e.g., excited-state-based photodetectors, all-optical modulators).

## Materials and methods

**Fig. 5:**
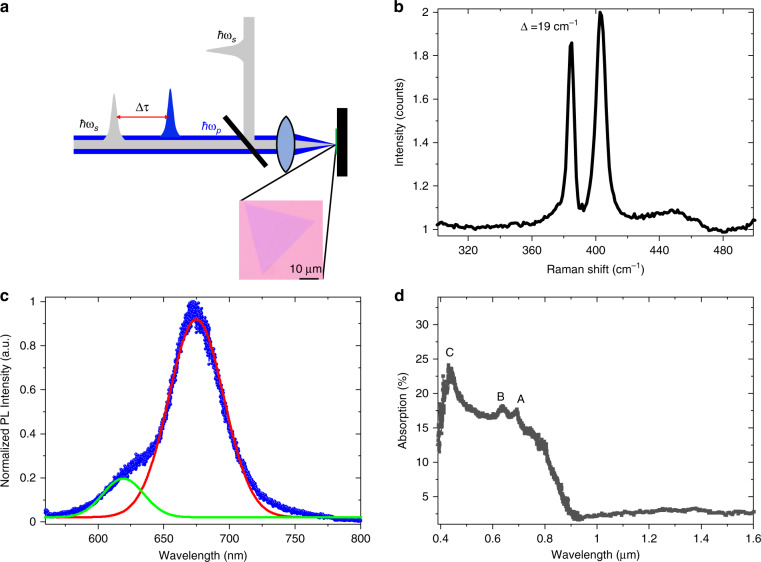
Experimental setup and sample characterization results. **a** Simplified experimental setup, with an optical image of a typical MoS_2_ flake on the SiO_2_/Si substrate shown in the inset. **b** Raman shift with excitation at a wavelength of 488 nm. The difference between the two Raman peaks (∆) is ~19 cm^−1^. **c** Normalized photoluminescence (PL) spectrum of a typical MoS_2_ flake with excitation at 400 nm. The fitted Gaussian curves show two prominent peaks, one at the A-exciton (~0.675 µm, red curve) and the other at the B-exciton (~0.62 µm, green curve). **d** Linear absorption spectrum of ML-MoS_2_, with absorption peaks at the “A”, “B”, and “C” exciton positions

We use an ultrafast pump-probe^47^ TA setup for the measurement (Fig. 5a). An optical parametric amplifier (Spectra-Physics, TOPAS) with a repetition rate of 2 kHz is used to generate the pump and probe light pulses. The pump light at 400 nm is achieved by frequency doubling of the 800 nm beam from the fundamental source of the optical parametric amplifier with a BBO crystal. The wavelength range of the probe beam comes from the optical parametric amplifier, which is tuneable from 0.86 µm to 1.4 µm. The pulse width of both the pump and probe light pulses is ~230 fs. After crossing a time delay line, the pump and probe lights are spatially merged using a dichroic mirror, and the combined beam is focused onto the sample with an objective (NA = 0.75, 40×). The spot sizes of the pump beam and probe beam on the sample are ~2.5 µm and ~2.2 µm, respectively. The reflected probe light is separated from the pump light using filters and measured with an infrared detector following a monochromator (Andor 328i).

Monolayer flakes of MoS_2_ are grown on a SiO_2_/Si substrate (SiO_2_ thickness of 285 nm) by the chemical vapor deposition (CVD) method^53^. ML-MoS_2_ is grown with ~10 mg of sulfur (at 170 °C) and an ~0.5/15 mg NaCl/MoO_3_ mixture (at 750 °C) for 5 min in high-purity argon. An optical microscopic image of ML-MoS_2_ is shown in the inset of Fig. 5a. The Raman spectrum of the sample, obtained using a continuous-wave laser at 488 nm, shows two peaks, $E_{2g}^1$ and *A*_1*g*_, at ~384 cm^−1^ and 403 cm^−1^, respectively, with a peak position difference (∆) of ~19 cm^−1^, as shown in Fig. 5b. Furthermore, the PL measurement shows two peaks, one at ~0.675 µm and another at 0.62 µm, corresponding to the A and B excitons, respectively, as shown in Fig. 5c, which is fitted with two Gaussian curves at the peak positions. The results show that the optical bandgap of our MoS_2_ sample is ~1.83 eV. Note that the Raman peaks and the PL spectra agree well with the previous results^10^, which confirms the high quality of our monolayer MoS_2_ flakes and their semiconducting 2H phase^54,55^. The linear absorption result from the visible to NIR range is shown in Fig. 5d. Strong absorption is observed at the “A”, “B”, and “C” exciton positions, as marked in Fig. 5d, whereas a flat absorption response of ~2–3% is observed in the NIR region. Considering the linear absorption of ML-MoS_2_ of ~20% at 400 nm, the exciton density with ~1 µW average power is estimated as ~7 × 10^15^ cm^−2^ assuming 100% efficiency of the exciton excitation. The intrinsic doping level of our CVD-grown ML-MoS_2_ is ~10^8^~10^9^ cm^−2^ by electrical transport measurement. Since the beam spot size is much smaller than the flake, we carefully avoid the influence of the edge-state by ensuring the spot always in the middle of the flake.

## Supplementary information

Supplementary Information
