# Supplementary material for: Ultrafast transient sub-bandgap absorption of monolayer MoS2
Source: Light Sci Appl. 2021 Jan 29;10:27. doi: 10.1038/s41377-021-00462-4 (PMC7846580; doi:10.1038/s41377-021-00462-4)
Supplement: Supplementary file 1 — Supplementary Information [file 41377_2021_462_MOESM1_ESM.docx]

**Supplementary Information**

**Ultrafast transient sub-bandgap absorption of monolayer MoS_2_**

Susobhan Das^1,*^, Yadong Wang^1^, Yunyun Dai^1^, Shisheng Li^2^, and Zhipei Sun^1, 3,*^

^1^Department of Electronics and Nanoengineering, Aalto University, Espoo 02150, Finland

^2^International Center for Young Scientists (ICYS), National Institute for Materials Science (NIMS), Tsukuba, Japan

^3^QTF Centre of Excellence, Department of Applied Physics, Aalto University, Espoo, Finland

*Emails: [Susobhan.das@aalto.fi](mailto:Susobhan.das@aalto.fi); [zhipei.sun@aalto.fi](mailto:zhipei.sun@aalto.fi)

1. **Theoretical Modeling**

**
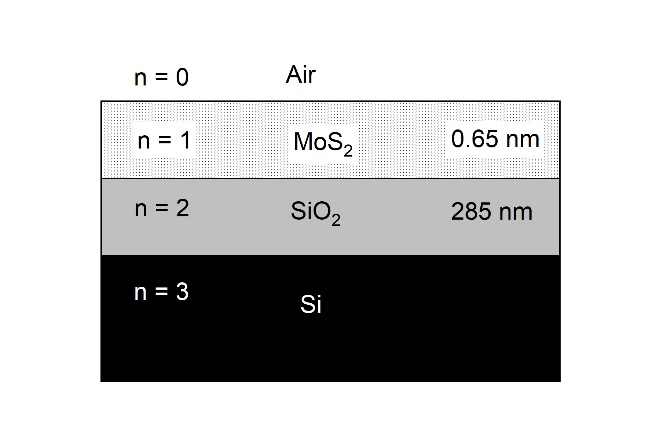
**

**Fig. S1:** Schematic of ML-MoS_2_ on a SiO_2_/Si substrate with thicknesses of corresponding layers.

To calculate the absorption of ML-MoS_2_, it is required to estimate the permittivity in the required wavelength. Considering both *A* and *B* exciton band transition, the total imaginary part of the permittivity can be expressed as Liu, Wang, Li and Liu ^1^

$$\varepsilon_{i}= \frac{f_{ex}^{A}\Gamma_{A}}{\left( E_{\omega}-E_{ex}^{A} \right)^{2}+\Gamma_{A}^{2}}+\frac{f_{ex}^{B}\Gamma_{B}}{\left( E_{\omega}-E_{ex}^{B} \right)^{2}+\Gamma_{B}^{2}}+\frac{f_{b}^{A}e}{\hbar\omega}\Theta\left( E_{\omega}-E_{g}^{A},\Gamma_{band}^{A} \right)+\frac{f_{b}^{B}e}{\hbar\omega}\Theta\left( E_{\omega}-E_{g}^{B},\Gamma_{band}^{B} \right)$$

where $\Gamma_{b}^{A}$($\Gamma_{b}^{B}$), $f_{ex}^{A}$($f_{ex}^{B}$), and $E_{ex}^{A}$($E_{ex}^{B}$) are the linewidth, equivalent oscillator strength, and transition energy of *A*(*B*) respectively. $f_{b}^{A}$($f_{b}^{B}$), $E_{g}^{A}$($E_{g}^{B}$) and $\Gamma_{band}^{A}$($\Gamma_{band}^{B}$) are equivalent oscillator strength of interband transition, bandgap, linewidth of the interband transition of *A*(*B*) band respectively. $E_{g}^{B}$ can be expressed as $E_{g}^{B}=E_{g}^{A}+\Delta_{band}$**.** $\Theta\left( x,\Gamma\right)=\frac{1}{\pi}\int_{-\infty}^{x} \frac{\Gamma}{\Gamma^{2}+\psi^{2}}d\psi$ is the step function with a broadening of $\Gamma$.

The real part of the permittivity of ML-MoS_2_ can be obtained using Kramers-Kroning relations

$$\varepsilon_{r}\left( \omega\right)=\varepsilon_{r0}+\frac{1}{\pi}\mathbb{p}\int_{0}^{\infty} \frac{s\varepsilon_{i}\left( s \right)}{s^{2}-\omega^{2}}ds$$

where $\mathbb{p}$ is the principle value integral and $\varepsilon_{r0}$ is the static dielectric constant tensor due to the optical anisotropy, for the normal incident of light, $\varepsilon_{r0}\approx4.2$. Note that, we do not consider the nonlinear absorption of ML-MoS_2_ in this simplified modeling.

The values of the fitted parameters are listed in Table 1.

| Table 1 | | | | | | | | | | | |
| --- | --- | --- | --- | --- | --- | --- | --- | --- | --- | --- | --- |
| $f_{ex}^{A}$ | $f_{b}^{A}$ | $\Gamma_{A}$ | $\Gamma_{band}^{A}$ | $E_{ex}^{A}$ | $f_{ex}^{B}$ | $f_{b}^{B}$ | $\Gamma_{B}$ | $\Gamma_{band}^{B}$ | $E_{ex}^{B}$ | $E_{g}^{A}$ | $\Delta_{band}$ |
| 0.29  eV | 74 | 28  meV | 244  meV | 1.887  eV | 0.40  eV | 50 | 46  meV | 82  meV | 2.03  eV | 2.60  eV | 146  meV |

Since, ML-MoS_2_ is on SiO_2_/Si substrate, to obtain the absorption in our structure, the transfer matrix method (TMM) is used^2^. For linearly polarized (along the x-axis) normal incident light, propagating along the z-direction, the total electric field in the n^th^ layer is given by

$$E_{n}\left( z \right)=\left[ A_{n}e^{-i\kappa_{nz}(z-z_{n})}+B_{n}e^{i\kappa_{nz}(z-z_{n})} \right]\hat{x}$$

where *A_n_* and *B_n_* are the amplitudes of forward and backward going electric field at n^th^ layer respectively. *k_nz_* is the complex wave vector and *z_n_* is the position of the n^th^ layer along the z-direction. Applying the boundary condition of each layer interface, the electric field amplitudes of (n+1)^th^ layer are related to the incident fields by transfer matrix

$$\left( \begin{matrix} A_{n+1} \\ B_{n+1} \end{matrix} \right)=\left( \begin{matrix} T_{11} & T_{12} \\ T_{21} & T_{22} \end{matrix} \right)\left( \begin{matrix} A_{0} \\ B_{0} \end{matrix} \right)$$

Therefore, the absorptance $\mathcal{A}_{0}$ of ML-MoS_2_ can obtain from the Poynting vector $\mathcal{A}_{0}=\frac{S_{oi}+S_{2i}+S_{0o}-S_{2o}}{S_{0i}}$ where *S_0i_* and *S_0o_* (*S_2i_* and *S_2o_*) are the incident and outgoing Poynting vectors in the air (in the SiO_2_ layer), respectively as shown in Fig. S1. Also, $S_{0i}=\beta_{0}A_{0}^{2},$ $S_{0o}=\beta_{0}B_{0}^{2}$, $S_{2i}=\beta_{2}B_{2}^{2}$, and $S_{2o}=\beta_{1}A_{2}^{2}$ where $\beta_{0}=\sqrt{{\varepsilon_{0}}/{\mu_{0}}}$, and $\beta_{2}=\sqrt{{\varepsilon_{{SiO}_{2}}}/{\mu_{0}}}$. Here $\varepsilon_{0}$ and $\mu_{0}$ are free space permittivity and permeability respectively and $\varepsilon_{{SiO}_{2}}$ is relative permittivity of SiO_2_.

The reflection from the ML-MoS_2_ flake is measured for the probe wavelength range with the same experimental setup as described in Fig. 5a of the main text. The probe power *P_s_* (intensity *I_s_*) is fixed ~1µW (~28.93 GW/cm^2^) for the whole wavelength range.

The measured reflectance from the sample and our theoretical prediction from modeling at our probe wavelength range are shown in Fig. S2 which reflects that our theoretical prediction has good agreement with the experimental measurement.


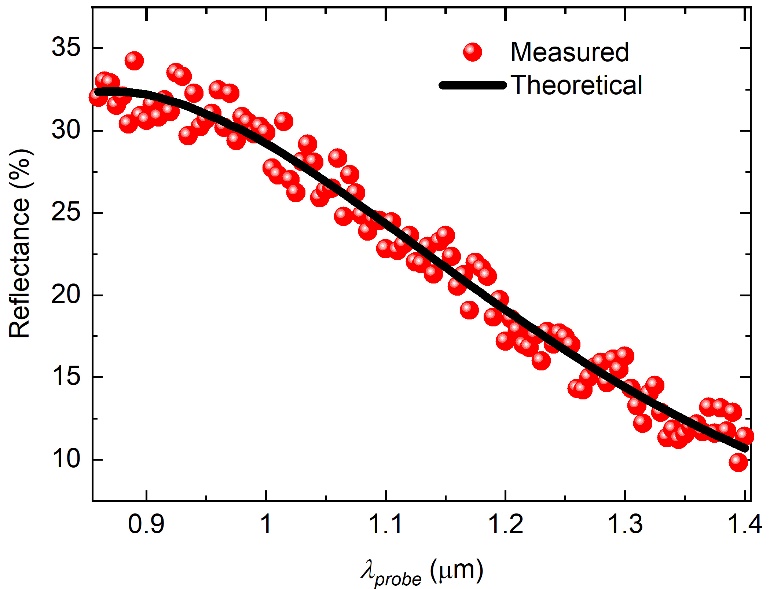


**Fig. S2:** Comparison between theoretical modeling and experimental results. The reflectance of ML-MoS_2_ on a SiO_2_/Si substrate where the solid black line is the theoretical prediction and red dots are experimentally measured.

In our experiment, we observe that due to the presence of pump light, the reflection from ML-MoS_2_ at the probe wavelengths is modulated. Since the imaginary part of the refractive index is responsible for the absorption, here we assume that, the real part of the refractive index of ML- MoS_2_ is not changing and the modulation is only caused by the change in imaginary part of its refractive index due to the presence of the pump light. Therefore, from the change in reflectance of ML- MoS_2_ with and without the pump light, we estimate the change in the imaginary part of ML- MoS_2_ refractive index and the absorption change simultaneously using TMM. As a demonstration of our calculation, we show the change in reflection (*∆R=(R*_1_-*R*_0_*)/R*_0_) from our measurement and the corresponding change in absorption (*∆A=A*_1_-*A*_0_) for two probe wavelengths at ~0.88 and 1.13 µm in Fig. S3.

**
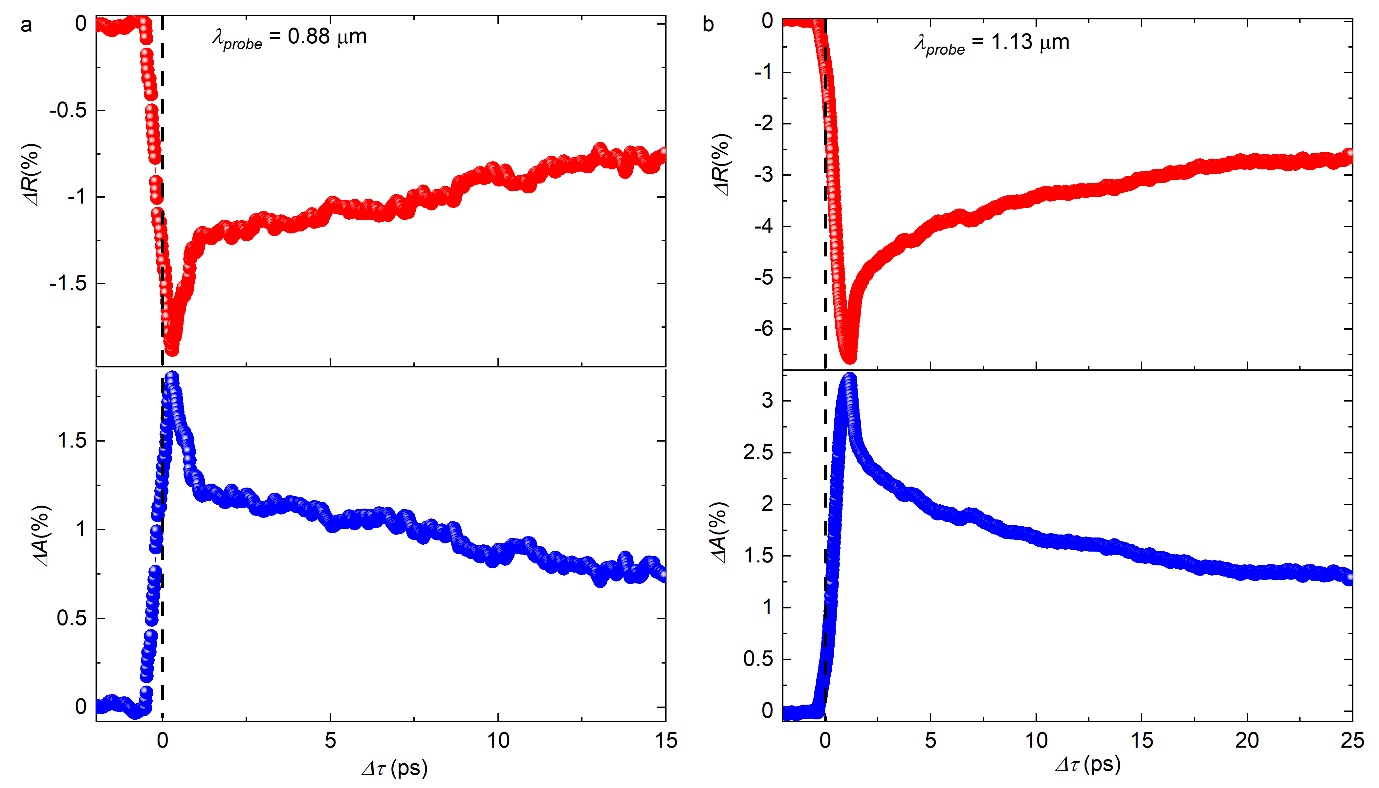
**

**Fig. S3:** Experimental measurement of change in reflection (*∆R=(R*_1_-*R*_0_*)/R*_0_) and corresponding change in absorption (*∆A=A*_1_-*A*_0_) from the theoretical model for probe wavelength (a) 0.88 µm and (b) 1.13 µm. *R_1_, A_1,_* are the reflection and absorption with the presence of pump and *R_0_, A_0,_* are the reflection and absorption without pump light.

1. **Time constants for transient absorption dynamics**

To evaluate the rising time constant of the dynamics, the single exponential function $y=ae^{-\frac{t}{\tau_{0}}}+c$ is used. Also, for decay dynamics, the bi-exponential function $y=ae^{-\frac{t}{\tau_{1}}}+be^{- \frac{t}{\tau_{2}}}+c$ is implemented to evaluate the fast decay and slow decay time constants. The fitting time constants over the probe wavelength range is shown in Fig. S4a. The rising graph at the shorter wavelength is very sharp, therefore single exponential function doesn’t fit very well. From the rise time constant of the longer wavelength range, we can predict that the value of the rise time constant (τ_0_) for the shorter wavelength is <300 fs.

Also, the rising start time ∆τ (increment = 1%) and the rise time (t_rise_) from (1% to 99%) are shown in Fig. S4b.


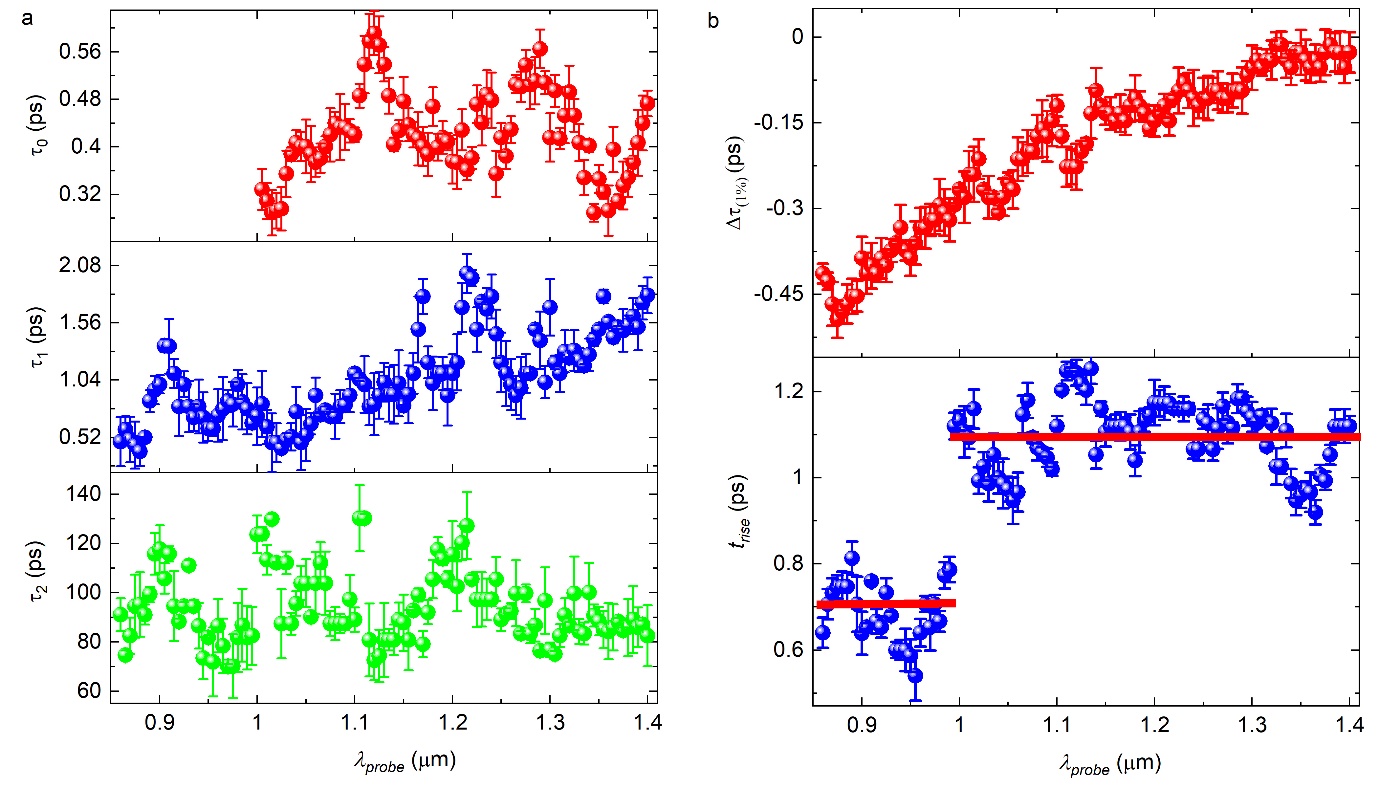


**Fig. S4:** (a) rise time constant (*τ_0_*), fast recovery time constant (*τ_1_*) and slow recovery time constant (*τ_2_*) of the transient absorption response over the probe spectral range. (b) The rising start time ∆τ (increment = 1%) is in the top panel and rise time (*t_rise_*) from (1% to 99%) is in the bottom panel; the average rise time is shown with the solid red line for both shorter and longer wavelength ranges.

1. **Power dependent transient absorption dynamics**

~~
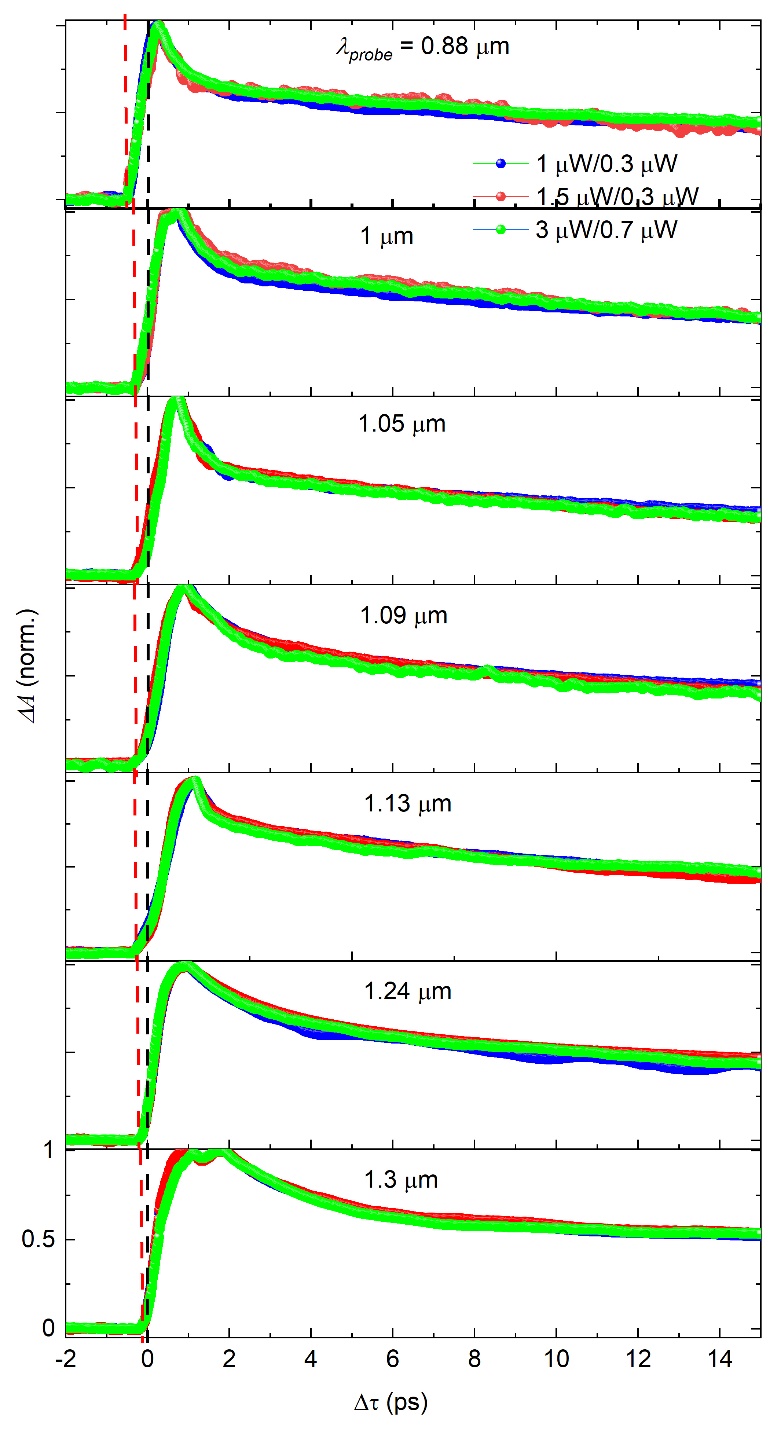
~~

**Fig. S5:** Normalized time-resolved absorption modulation at few selected probe wavelengths with different probe/pump power levels. Black dashed line indicates zero delay time position. Red dashed line indicates the starting point that the absorption begins to increase.

1. **TPA measurement with open aperture z-scan**

CVD growth MoS_2_ sample is transferred on a transparent sapphire substrate for performing open aperture z-scan measurement. The measurement has done at the probe wavelength 0.92 µm with *P_s_ ~10 µW*. From mathematical fitting^3^ we estimated that the TPA coefficient is $\sim5.83\times{10}^{3} cm/GW$.

**
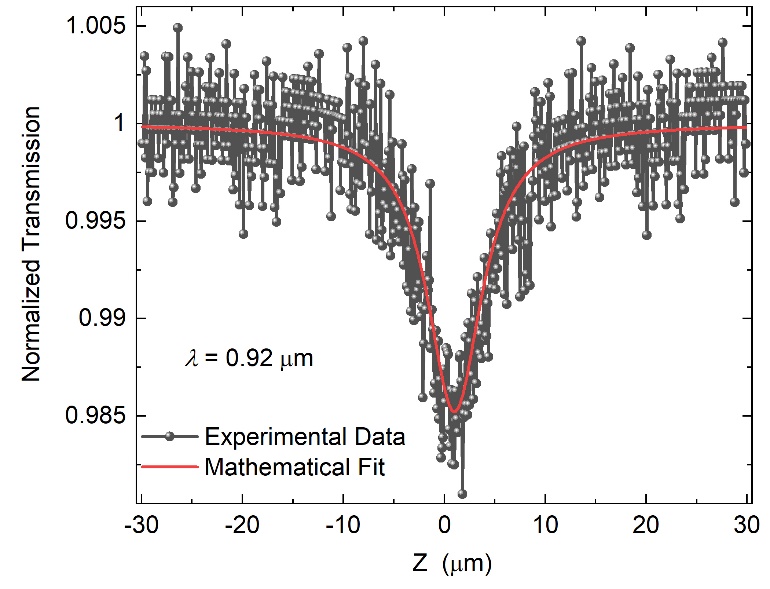
**

**Fig. S6:** Normalized transmission measurement of MoS_2_ flake at 0.92 µm probe wavelength with z-scan distance. Solid red curve is the mathematical fitting of the experimental results.

1. **Transient absorption dynamics of ML-WS_2_**

The transient absorption measurement on ML-WS_2_ shows similar phenomena, where the shorter probe wavelength (~0.92 µm) has a shorter rise time and the longer wavelength (~1.19 µm) takes a much longer time. As the electrical bandgap of ML-WS_2_ is ~2.41 eV^4^, we are expecting the boundary between shorter and longer wavelengths will be at ~1.03 µm. This conclude the different rising dynamics of transient absorption on ML-WS_2_ as shown in Fig. S7 confirms our model. Other monolayer 2D materials will be measured for full confirmation.


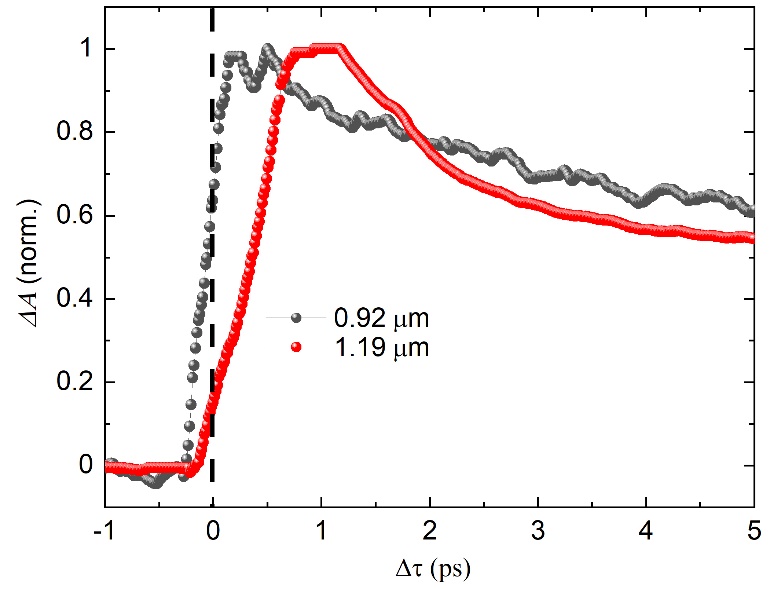


**Fig. S7:** Normalized TA modulation (∆A) of WS_2_ at probe wavelengths of 0.92 µm (black), and 1.19 µm (red). Black dashed line indicates zero delay position.

References

1. Liu, J.-T., Wang, T.-B., Li, X.-J. & Liu, N.-H. Enhanced absorption of monolayer MoS_2_ with resonant back reflector. *Journal of Applied Physics* **115**, 193511 (2014).

2. Liu, J.-T., Liu, N.-H., Li, J., Li, X.J. & Huang, J.-H. Enhanced absorption of graphene with one-dimensional photonic crystal. *Applied Physics Letters* **101**, 052104 (2012).

3. Li, Y. et al. Giant two‐photon absorption in monolayer MoS_2_. *Laser & Photonics Reviews* **9**, 427-434 (2015).

4. Chernikov, A. et al. Exciton Binding Energy and Nonhydrogenic Rydberg Series in Monolayer WS_2_. *Physical Review Letters* **113**, 076802 (2014).
